# Time-Course Evaluation of Body Mass Index in Japanese Children With Obstructive Sleep Apnea Syndrome After Adenotonsillectomy: A Three-Years Follow-Up Study

**DOI:** 10.3389/fped.2020.00022

**Published:** 2020-02-04

**Authors:** Ken Fukuda, Hiroki Yasudo, Naoki Ohta, Hiroko Narumi, Nozomi Abe, Shunsuke Tarumoto, Hiroshi Yamashita, Kiyoshi Ichihara, Shouichi Ohga, Shunji Hasegawa

**Affiliations:** ^1^Department of Pediatrics, Yamaguchi University Graduate School of Medicine, Ube, Japan; ^2^Department of Otolaryngology, Yamaguchi University Graduate School of Medicine, Ube, Japan; ^3^Department of Laboratory Sciences, Faculty of Health Sciences, Yamaguchi University Graduate School of Medicine, Ube, Japan; ^4^Department of Pediatrics, Kyushu University Graduate School of Medical Science, Fukuoka, Japan

**Keywords:** body mass index, pediatrics, obstructive sleep apnea syndrome, adenotonsillectomy, growth improvement

## Abstract

Delayed physical growth is a common complication of pediatric obstructive sleep apnea syndrome (OSAS). Adenotonsillectomy (AT) is the first-line treatment for pediatric OSAS. Only a few studies have performed time-course BMI evaluation in pediatric OSAS patients post-operatively. Thus, we aimed to evaluate the time-course changes in pediatric OSAS patients after AT. Thirty-three children with OSAS who underwent AT were included and divided into two groups on the basis of their BMI z-scores (delayed physical growth group, *n* = 15; non-delayed physical growth group, *n* = 18). Clinical records of height and weight were collected before AT and at 6, 12, 24, and 36 months after AT. Changes in the mean BMI z-scores of the two groups were assessed up to 36 months. The mean BMI z-score was significantly increased in the delayed physical growth group at 6 months after AT. In contrast, the increase in mean BMI z-score was not observed in the non-delayed physical growth group. Growth improvement was noted in pediatric OSAS patients with delayed physical growth after AT. Our results suggest that AT is a promising therapy for improving the physical growth of pediatric OSAS patients with such problems.

## Introduction

Obstructive sleep apnea syndrome (OSAS) in children is defined as a breathing disorder that occurs during sleep, characterized by sustained partial upper airway obstruction and/or intermittent complete obstruction (obstructive apnea), that inhibits normal breathing during sleep (1). It can occur in children of all ages from neonates to adolescents, and its prevalence is at least 1–3% (1–3). The clinical characteristics are snoring, difficulty in breathing during sleep, and repeated obstructive apnea (1, 4). Adenotonsillar hypertrophy (ATH) is the primary cause of OSAS in children, resulting in intermittent pharyngeal airway collapse intervened by airflow, hypoxemia, hypercapnia, and brief arousals from sleep (4). Complications of OSAS in children may include nocturnal enuresis, sweating during sleep, reduced neurocognition, behavioral problems, and school performance decline (5–7). Among the complications of OSAS, delayed physical growth is one of the most common concerns (5, 8, 9). Bonuck et al. (9) estimated that the prevalence of delayed physical growth due to OSAS in children younger than 6 years was 21%. ATH and sleep-disordered breathing are risk factors in the etiology of growth failure.

The first-line treatment of pediatric OSAS is adenotonsillectomy (AT). Polysomnographic resolution after AT is associated with symptom resolution (5, 10–13). Many studies have shown increases in height, weight, and BMI in OSAS patients after AT (5, 13–23), suggesting physical growth improvement. However, most of the studies measured patients' BMI only at a certain time point after the operation or lacked full records of time-course evaluation of BMI after the operation, indicating that the evaluations in these studies might be insufficient owing to the limited measurements of BMI for the time-course analysis of physical growth improvement after the operation. Additionally, the effect of AT should be compared between OSAS patients with and without delayed physical growth, as there are no studies investigating the effects of AT in OSAS patients according to the presence or absence of delayed physical growth.

The aim of our study was to evaluate physical growth changes in pediatric OSAS patients by consistently measuring BMI z-scores at 6, 12, 24, and 36 months after AT and to investigate how AT could affect physical growth changes in pediatric OSAS patients with or without delayed physical growth.

## Materials and Methods

### Participants

**Figure 1:**
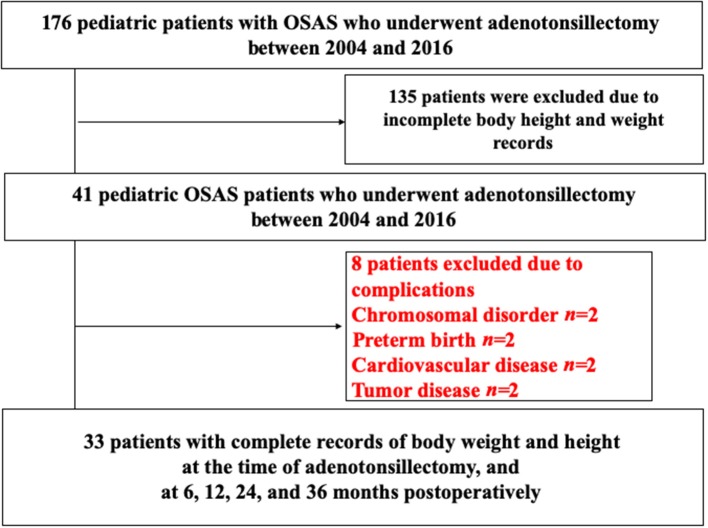
Flow chart for selecting the OSAS patients in this study. OSAS, obstructive sleep apnea syndrome.

This is a retrospective observational study. We consecutively accumulated 176 OSAS children who underwent AT between January 2004 and May 2016 at the Department of Pediatrics and Otolaryngology in Yamaguchi University Hospital (24). OSAS was diagnosed on the basis of the International Classification of Sleep Disorders (third edition). Eight children were excluded because of comorbidity of chromosomal disorders, preterm birth, other cardiovascular diseases, and tumorous diseases. Of the remaining 168 patients, 135 were excluded because of incomplete body height and weight data within the required observation period of 36 months. As a result, 33 pediatric patients were enrolled in this study as shown in the flow chart of the case selection process (Figure 1).

Before the start of the operation, informed consent was obtained from patients and patients' parents. The study design was approved by the institutional ethics committee of Yamaguchi University Hospital (No. H30-150). For safety reasons, eight patients were excluded from the study if they had craniofacial anomalies, obesity, Down syndrome, neuromuscular disorders, sickle cell disease, mucopolysaccharidoses, cardiac complications, current respiratory infections, and a history of prior upper airway surgery. As a result, 33 pediatric OSAS patients with completely body weight and height records at the time of AT and at 6, 12, 24, 36 months post-operatively were enrolled for the study.

The enrolled patients were divided into two groups according to the values of the BMI z-scores: the delayed physical growth group (BMI z-scores <0, *n* = 18) and the non-delayed physical growth group (BMI z-scores ≥0, *n* = 15) to determine if there is any difference in the changes of the BMI z-score values after the operation.

### Outcome Measures

We retrospectively analyzed the hospital records of OSAS patients who underwent AT at the Department of Otolaryngology and were followed up for 3 years post-operatively. All subjects were measured height and weight preoperatively and at 6, 12, 24, and 36 months post-operatively. All measurements were conducted by trained nurses. Height was measured by using a stadiometer to the nearest 1.0 mm. Weight was measured by using an electronic scale to the nearest 0.1 kg. For each child, z-scores for height and weight values were calculated by using the file produced by the Japanese 2,000 references (25). BMI z-scores were calculated according to Inokuchi et al. (26).

### Statistical Analysis

All statistical analyses were performed using JMP®13.1 (SAS Institute Inc., Cary, NC, USA). Data were analyzed for normal distributions using the Kolmogorov–Smirnov test and were presented as mean ± standard deviation. Pre-operative and post-operative (up to 24 months) SDS values for height and weight were compared using the paired *t*-test. To determine the significance of time-dependent changes between the pre-operative and post-operative (up to 36 months) BMI z-scores, the paired *t*-test was used with Bonferroni correction for multiple testing.

## Results

### Baseline Growth Characteristics of Pediatric OSAS Patients

We evaluated the characteristics of the pediatric patients to determine any presence of delayed physical growth (Table 1). The mean height z-score, weight z-score, and BMI z-score at the operation were −0.42 SD (±1.45), −0.45 SD (±1.31), and −0.12 SD (±1.99), respectively, indicating that OSAS patients who underwent AT tend to have overall delayed growth.

**Table 1:** Clinical characteristics of OSAS patients.

	**All: *n* = 33**	**Non-delayed physical growth group: *n* = 15**	**Delayed physical growth group: *n* = 18**	***P*-value**
Female/male ratio (*n*)	0.57(12/21)	0.50(5/10)	0.63(7/11)	0.74
Age (month)	55.5(±16.1)	57.9(±20.5)	53.6(±11.5)	0.44
Height (cm)	100.8(±12.0)	105.9(±15.7)	99.2(±6.91)	0.07
Height z-score	−0.42(±1.45)	0.07(±1.72)	−0.89(±1.04)	0.04
Body weight (kg)	15.7(±5.60)	20.0(±6.89)	14.3(±2.07)	<0.001
Body weight z-score	−0.45(±1.31)	0.62(±1.41)	−0.86(±0.74)	<0.001
BMI	15.4(±1.99)	17.4(±1.80)	14.5(±0.87)	<0.001
BMI z-score	−0.12(±1.48)	1.12(±1.02)	−1.14(±0.91)	<0.001

*OSAS, obstructive sleep apnea syndrome; BMI, body mass index*.

### Physical Growth Difference After at Between the Delayed and Non-delayed Physical Growth Groups

**Figure 2:**
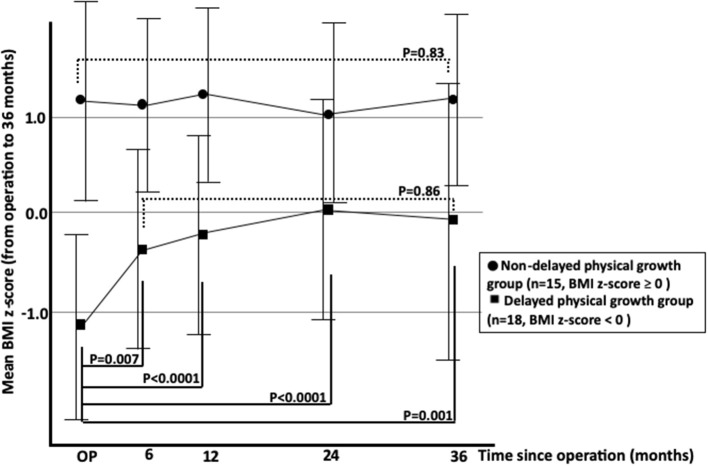
Changes in mean BMI z-scores of OSAS patients with delayed and non-delayed physical growth after adenotonsillectomy. OSAS, obstructive sleep apnea syndrome; BMI, body mass index.

There were no differences in the ratio of male to female and age between the two groups (Table 1). Moreover, we compared the mean BMI z-scores preoperatively and at 6, 12, 24, and 36 months after AT between the two groups (Figure 2). The mean BMI z-score had significantly increased by 6 months after AT in the delayed physical growth group, although there were no significant differences between 6 and 36 months after AT. Conversely, the mean BMI z-score had not increased in the non-delayed physical growth group during the study period. These results suggest that physical growth recovery was observed in the delayed physical growth group but not in the non-delayed physical growth group.

**Figure 3:**
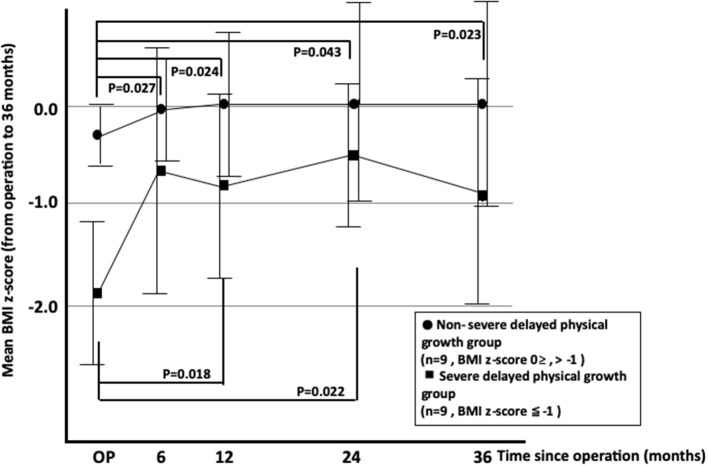
Changes in mean BMI z-scores of OSAS patients with severe and non-severe delayed physical growth after adenotonsillectomy. OSAS, obstructive sleep apnea syndrome; BMI, body mass index.

We further divided the delayed physical growth group into two subgroups based on the values of BMI z-score [non-severe subgroup (*n* = 9): BMI z-score 0≥ and −1.0<; severe subgroup (*n* = 9): BMI z-score ≦ −1.0] (Figure 3). BMI z-scores had increased in both subgroups at 6 months after AT (Figure 3). However, changes in BMI z-score were more drastic in the severe subgroup than in the non-severe subgroup, suggesting that physical growth recovery was more prominently observed in patients with severe delayed physical growth at 6 months after AT than in those without severe delayed physical growth.

## Discussion

In this study, we compared the physical growth after AT between pediatric OSAS patients with and those without delayed physical growth. We found that the mean BMI z-score in patients with delayed physical growth was significantly increased after the operation and returned to the normal levels, whereas in patients without delayed physical growth, the mean BMI z-score did not increase. Additionally, the mean BMI z-score was more drastically increased in patients with severe delayed physical growth than in those without severe delayed physical growth. These results suggest that growth recovery can be expected in OSAS patients with delayed physical growth. Additionally, more dramatic physical growth recovery can be expected within 6 months in subjects with severe delayed physical growth after the AT operation.

Several lines of evidence have revealed that there are some factors associated with delayed physical growth in OSAS. OSAS patients are reported to be insensitive to growth hormone (GH) releasing hormone-induced GH response and a delayed insulin-like growth factor type 1 (IGF-1) synthesis after GH injection in untreated disease (27). AT may restore the GH axis activity resulting in growth recovery. Moreover, hypertrophied tonsil is a mechanical barrier for food intake, thus reducing caloric intake (28). Bar et al. (19) showed that 83% of parents reported an increase in their children's appetite following AT. Nachalon et al. (28) indicated a significant change in diet composition in children with OSAS after surgery. Sleep energy expenditure reduction and weight gain improvement by surgery suggest that the increased energy expenditure could have been caused by increased respiratory work during sleep in OSAS children (14). The reasons for physical growth recovery after AT in OSAS patients were not fully understood. However, the imbalance between caloric intake, energy expenditure, and insufficient growth hormone secretion could be associated with physical growth changes after AT. It is reported that IGF-1 and IGF binding protein-3 levels increase 3–6 months after AT (19, 24, 29). These results are consistent with the timing of physical growth recovery observed in our study.

There are some studies reporting obesity resulting from AT in OSAS patients (17, 20–22). In our study, the mean BMI z-score increased and returned to normal levels in OSAS patients with delayed physical growth, whereas the BMI z-score was not increased in those without delayed physical growth up to 36 months after AT. These results suggest that obesity is unlikely to be caused by AT in OSAS participants, regardless of the presence of delayed physical growth. The prevalence of obesity in Japanese 5 years-old is 2.73% for boys and 2.58% for girls (30). Asian children are more susceptible to metabolic disorders caused by obesity of even mild degree than the Caucasian. For this reason, more attention is generally paid to obesity at an early stage. Therefore, obese children may have already been restricted of food intake at home or school prior to the surgery, which may have suppressed the increase in post-operative food intake. As a matter of fact, a Korean report (31), which enrolled OSAS children similar to those of our study, had the same trend as our results. Hence, the peculiar phenomenon may be caused by racial difference. This study focused on delayed physical growth subgroup, but we would like to increase the number of cases so that the subgroup of obesity could be evaluated in the future.

Although there are many reports mentioning delayed physical growth as a common complication of OSAS, it is difficult to tell whether it is directly caused by OSAS or other factors. Our results showed that the mean BMI z-score returned to normal levels in participants with delayed physical growth after AT and that the mean BMI z-score was more drastically increased in patients with severe delayed physical growth within 6 months after AT. These results suggest that delayed physical growth in OSAS patients could be directly caused by OSAS, not by other reasons, and that AT can be a promising therapy to improve delayed physical growth in OSAS patients.

There are some limitations to this study. This study is a pilot study conducted in a single center. Further multi-center or prospective studies involving different regions, races, and populations are warranted to confirm whether AT is a promising therapy for growth improvement in OSAS patients with such problems.

## Conclusions

Physical growth improvement was observed within 6 months after AT in OSAS patients with delayed physical growth but not in those without delayed physical growth. Thus, AT is a promising therapy for improving the physical growth of OSAS patients with such problems.

## Data Availability Statement

All datasets generated for this study are included in the article/supplementary material.

## Ethics Statement

The studies involving human participants were reviewed and approved by the Institutional Ethics Committee of Yamaguchi University Hospital. Written informed consent to participate in this study was provided by the participants' legal guardian/next of kin.

## Author Contributions

HYas, SO, and SH were the principal investigators taking primary responsibility for the manuscript. KF, HYas, KI, HYam, SO, and SH performed the clinical management with helpful discussion for the completion of the study. KF, NO, HN, NA, ST, and HYam took responsibility for the diagnosis and data collection.

### Conflict of Interest

The authors declare that the research was conducted in the absence of any commercial or financial relationships that could be construed as a potential conflict of interest.
